# Supplementary material for: Patterns of Intron Gain and Loss in Fungi
Source: PLoS Biol. 2004 Nov 30;2(12):e422. doi: 10.1371/journal.pbio.0020422 (PMC532390; doi:10.1371/journal.pbio.0020422)
Supplement: Table S1 — Also available at http://genes.mit.edu/NielsenEtAl/. (4.3 MB ZIP). [file pbio.0020422.st001.zip › NielsenEtAl/html/39.html]

AN0089.1.NCU03711.1.MG08144.1.FG05141.1


```
 CLUSTAL W (1.82) Multiple Sequence Alignments - Introns Inserted


Sequence 1: NCU03711.1	205 aa
Sequence 2: MG08144.1	205 aa
Sequence 3: FG05141.1	205 aa
Sequence 4: AN0089.1	201 aa
Alignment Length: 205 aa
Number Identitical Residues: 172 aa
Alignment Score (without introns) 6911


MG08144.1 	MSSRKKVLLK0VIILGDSGVGKTSLMNQY0VNKKFSASYKATIGADFLTREVLVDDRQVT
NCU03711.1	MSSRKKVLLK0VIILGDSGVGKTSLMNQY0VNKKFSASYKATIGADFLTREVLVDDRQVT
FG05141.1 	MSSRKKVLLK0VIILGDSGVGKTSLMNQY0VNKKFSASYKATIGADFLTREVLVDDRQVT
AN0089.1  	MSSRKKVMLK0VIILGDSGVGKTSLMNQY0VNKKFSGSYKATIGADFLTKEVLVDDR---
          	*******:** ****************** ******.************:*******   

MG08144.1 	MQ0LWDTAGQERFQSLGVAFYRGADCCVLVFDVNNSKSFDALDSWRDEFLIQASPRDPDN
NCU03711.1	MQ0LWDTAGQERFQSLGVAFYRGADCCVLVYDVNNSKSFDALDSWRDEFLIQASPRDPDN
FG05141.1 	MQ0LWDTAGQERFQSLGVAFYRGADCCVLVYDVNNAKSFEALDSWRDEFLIQASPRDPPN
AN0089.1  	-L0IWDTAGQERFQSLGVAFYRGADCCVLVYDVNNSKSFEALDSWRDEFLIQASPRDPES
          	   :**************************:****:***:****************** .

MG08144.1 	FPF0VVLGNKIDVEESKRV0ISTKRAMTFCQSKGGIPYFETSAKEAINVEQAFEV1IARN
NCU03711.1	FPF~VVLGNKIDMEESKRV0ISTKRAMTFCQSKGNIPYFETSAKEAINVEQAFEV~IARN
FG05141.1 	FPF0VVLGNKIDVEESKRV0ISNKRAMTFCQSKGDIPYFETSAKEAINIDQAFEV1IARN
AN0089.1  	FPF0VVIGNKIDMEESKRM~ISSKRAMTFCQSKGNIPYFETSAKEAVNVEQAFEV1IARS
          	*** **:*****:*****: **.***********.***********:*::***** ***.

MG08144.1 	ALAQEESEEFSGDFQDPINIHIDNDRDGCAC
NCU03711.1	ALMQEESEEFSGDFQDPINIHIENDRDGCAC
FG05141.1 	ALAQEESEEFSGDFDDPINIHIENDRDGCAC
AN0089.1  	ALAQEEAEEYGGDYTDPINIHDTTERDGCAC
          	** ***:**:.**: ******  .:******
```
